# Co-occurrence of myeloid neoplasm and plasma cell neoplasm

**DOI:** 10.4322/acr.2021.393

**Published:** 2022-07-21

**Authors:** Husam Jum’ah, Yan Wang, Salman Ayub

**Affiliations:** 1 MetroHealth Medical Center, Department of Pathology, Cleveland, Ohio, USA

**Keywords:** Myelodysplastic Syndromes, Monoclonal Gammopathy of Undetermined Significance, Smoldering Multiple Myeloma, Bone Marrow, Pancytopenia

## Abstract

Co-occurrence of myelodysplastic syndrome (MDS) and plasma cell neoplasm in patients with no history of chemo and/or radiotherapy is rarely reported. Herein, we report a case of a female in her seventieth decade of life who was referred to the hospital for pancytopenia. The patient was asymptomatic and was doing well overall. Serum protein electrophoresis was remarkable for a lambda-restricted monoclonal protein (IgG) estimated at 1.8g/dL. Immunoglobulin G serum level was also elevated, and serum Kappa/Lambda free light chain ratio was decreased. At that time, a bone marrow biopsy showed myelodysplastic syndrome with excess blasts-2 (MDS-EB2) and a monoclonal plasma cell proliferation. Some studies have shown that patients with plasma cell neoplasm could be associated with an increased risk of developing MDS compared to the general population. Based on reviewing the literature, to our knowledge, the pathological mechanism of the co-occurrence of both diseases is not yet clear.

## INTRODUCTION

MDS is a clonal disorder of the hematopoietic stem cells associated with cytopenia(s), dysplasia, ineffective bone marrow hematopoiesis, a tendency to develop acute myeloid leukemia, and in many cases, recurrent genetic abnormalities.^1^^-^ 4 Non-IgM Monoclonal Gammopathy of Undetermined Significance (MGUS) is associated with serum monoclonal immunoglobulin concentration of less than 30 g/L, bone marrow with < 10% light chain restricted plasma cells, and absence of end organ damage such as anemia, bone lesions, hypercalcemia, renal insufficiency or amyloidosis.^5^ Plasma cell myeloma diagnosis depends on a mixture of clinical, pathological, and radiological data. It is defined as the neoplastic proliferation of plasma cells associated with M protein and evidence of plasma cell neoplasm with end-organ damage (hypercalcemia, renal insufficiency, anemia, bone lesions).^6^^,^ 7 Smoldering myeloma is the stage between MGUS and plasma cell myeloma and is diagnosed by two criteria: (i) serum monoclonal protein (IgG or IgA) of 30 g/L or more, or urinary monoclonal protein of 500 mg/24 hours and/or bone marrow with 10-60% light chain restricted plasma cells proliferation, and (ii) the absence of myeloma defining events or amyloidosis.^5^

Chemotherapy Induced MDS is associated with multiple myeloma treatment, specifically melphalan.^8^^,^ 9 However, a few published articles support the association between MDS and/or acute myeloid leukemia (AML) with non-treated plasma cell neoplasm.^10^^-^ 12 The exact underlying mechanism of this co-occurrence has not been described yet.^10^^-^ 12 This paper aims to support further the possibility of the pathological association between MDS and plasma cell neoplasm.

## CASE REPORT

An asymptomatic female in her seventieth decade of life was referred to the hematologist because of pancytopenia, which was incidentally detected. Her medical history is remarkable for hyperlipidemia, essential hypertension, and atopic dermatitis. The patient does not drink alcohol. She was never submitted to chemotherapy or radiotherapy before. Her blood workup showed the hemoglobin of 7.8 g/dL (RR= 12.0 - 15.0 g/dL), white blood cells count of 4 K/uL (RR= 4.5 - 11.5 K/uL), platelets count of 109 K/uL (RR= 150 - 400 K/uL), elevated serum immunoglobulin-G level of 2,564 mg/dL (RR= 768 - 1,632 mg/dL), decreased serum Kappa/Lambda free light chain ratio of 0.22 (RR= 0.26 - 1.65), and mildly elevated serum creatinine of 1.15 mg/dL (RR= 0.50 - 1.10 mg/dL). Serum protein electrophoresis was remarkable for lambda-restricted monoclonal protein (IgG) of 1.8 g/dL. Urine protein electrophoresis, serum calcium, vitamin-B12, thyroid, hepatic function tests, and iron profiles were normal. No lytic lesions were identified on the skeletal survey.

**Figure 1:**
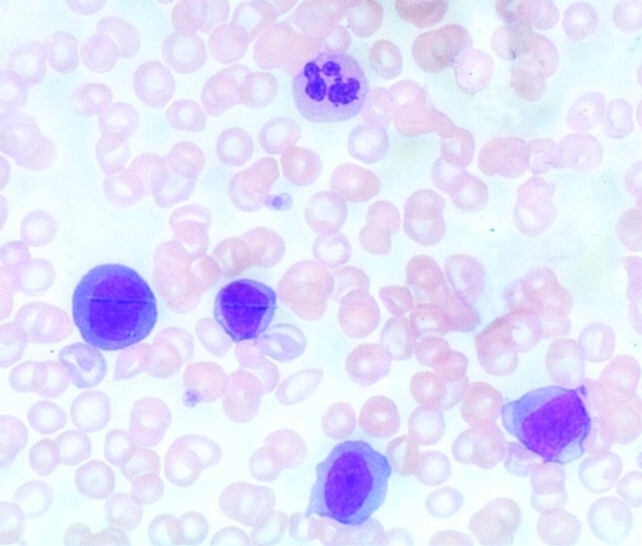
Peripheral blood smear with immature mononuclear cells with a moderate blue cytoplasm, some of them suspicious for blasts (hematoxylin and eosin stain, X60).

The peripheral blood smear showed 37% immature mononuclear cells with a moderate amount of dark blue cytoplasm; some were blasts (Figure 1).

**Figure 2:**
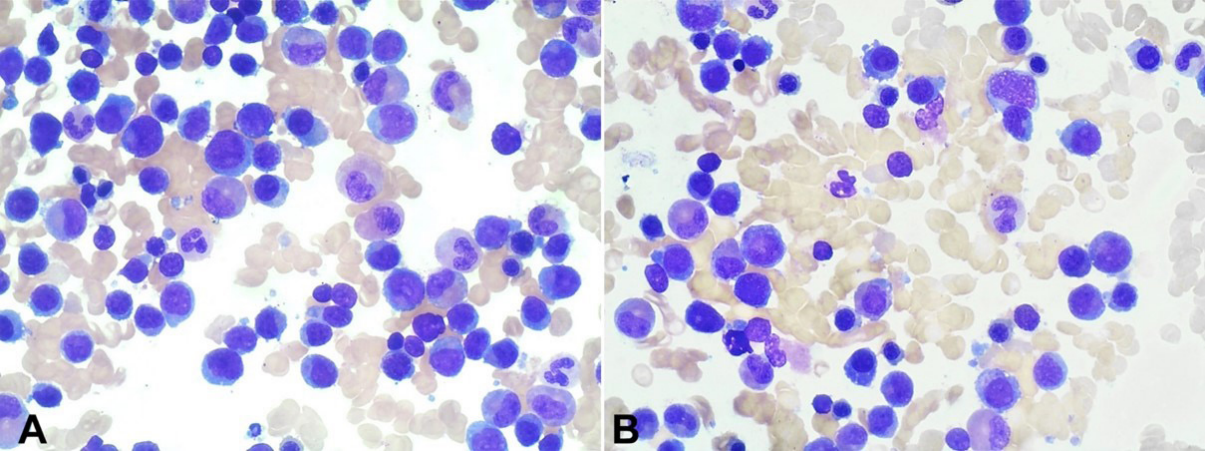
Bone marrow aspirate smear with some myeloblasts and immature cells suggestive of promyelocytes/ myelocytes but possibly representing blasts with atypical morphology. The picture also shows increased plasma cells (hematoxylin and eosin stain, 60X).

**Figure 3:**
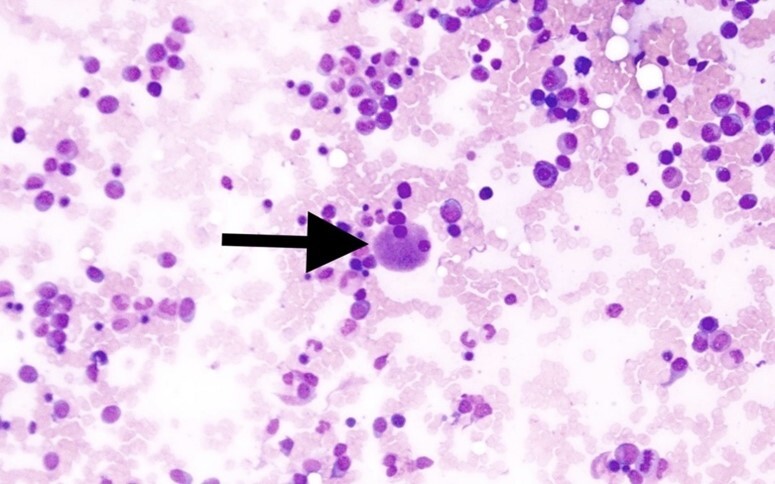
Bone marrow aspirate smear with a dysplastic megakaryocyte (arrow) (H&E, 40X).

The differential count on the bone marrow aspirate smear showed 15% myeloblasts. There were additional 24% immature cells suggestive of promyelocytes but possibly representing blasts with atypical morphology (Figures 2A and 2B), and more than 10% dysplastic megakaryocytes (Figure 3). Bone marrow flow-cytometry was remarkable for 12% of myeloblasts (positive for CD13, CD15, CD33, CD34, CD117 and HLA-DR, and negative for CD7, CD14, and CD19). Although the percentage of CD34+ cells is lower than 20%, it does not rule out that more immature cells are present in the aspirate smear.

**Figure 4:**
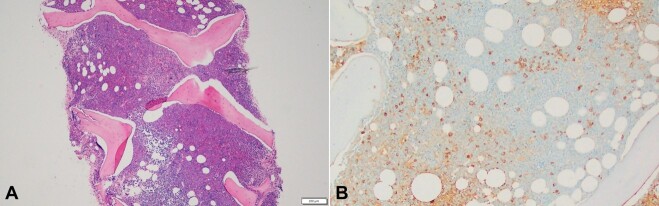
**A –** Hypercellular bone marrow core biopsy (~70% cellularity) (hematoxylin and eosin stain, 4 X); **B –** Bone marrow core biopsy immunohistochemistry for lambda shows increased lambda-restricted plasma cells (about 10% of cellularity) (10 X).

In addition, the bone marrow aspirate smear showed increased plasma cells (8% on manual differential count) (Figures 2A and 2B). Bone marrow core biopsy was hypercellular (70% of cellularity) (Figure 4A) with increased lambda-restricted plasma cells (about 10% of cellularity) (Figure 4B; immunohistochemistry for lambda). Bone marrow flow-cytometry was also remarkable for 3% lambda restricted plasma cells. Molecular studies show the following alterations: 2-FLT3 L601_K602insNFREYEYDL and ASXL1 E635fs*15. Chromosome analysis revealed a normal karyotype with no acquired clonal abnormality. The diagnosis of MDS-EB2, highly suspicious for AML and concurrent plasma cell neoplasm (clinically classified as smoldering myeloma) was made.

## DISCUSSION

This patient had pancytopenia, and on the aspirate smear, the megakaryocytes were decreased in number, and all of them showed dysplastic forms with separated nuclei. Also, his bone marrow flow-cytometry showed 12% myeloblasts, and differential count on peripheral blood smear showed 37% immature mononuclear cells, some of them were blasts, supporting the diagnosis of MDS-EB2 (highly suspicious for AML). In addition, on the core biopsy, there were lambda-restricted plasma cells, constituting about 10% of the total cellularity. The patient also had monoclonal protein (IgG lambda at 1.8 g/dL). We could not tell if the anemia (hemoglobin of 7.8 g/dL) resulted from plasma cell neoplasm-associated end-organ damage or if it was part of the pancytopenia caused by the myelodysplastic syndrome. Clinically, the plasma cell neoplasm was classified as smoldering myeloma.

Co-existing chemotherapy-induced MDS with multiple myeloma is well described. Melphalan, one of the drugs that can be used in treating multiple myeloma, has been involved in the pathogenesis of MDS in those patients.^8^^,^ 9 However, very few studies and reported cases support the association between MDS or MDS-AML and non-treated plasma cell neoplasms.^10^^-^ 12 Yoshida et al.,^11^ reported a case series of 14 patients with monoclonal gammopathy with concurrent MDS, which were unrelated to therapy. Of the 14 patients, twelve had monoclonal gammopathy of undetermined significance (MGUS), and two had smoldering myeloma. MGUS was found in all MDS subtypes of WHO classification n their case series. Given the lack of prior history of chemotherapy in all of their cases, they proposed that the co-existing presentation of MDS and plasma cell neoplasm suggested a true co-occurrence of both diseases. Our patient had never had chemotherapy, which supports that there might be a common pathological pathway for both diseases.

In our case, we do not know which disease preceded the other, the plasma cell neoplasm or the myelodysplastic syndrome. Roeker et al.,^10^ studied the risk of MDS and AML in patients with MGUS. The authors used a large population of 605 patients with MGUS and 16,710 control patients who were negative for the presence of a monoclonal protein. The authors found that MGUS is associated with an increased risk of developing MDS, but no statistically significant data suggested any association between MGUS and AML. Mailankody et al.,^13^ studied 8,740 multiple myelomas (MM) patients and 5,652 MGUS patients, and they found that MM patients have an 11.51-fold increased risk of AML/MDS and that MGUS had an 8.01-fold increased risk of AML/MDS. The increased risk in this study was only noticed in IgG and IgA MGUS patients but not in IgM; this finding supports a common pathogenic pathway between plasma cell neoplasms and AML/MDS.

It has been suggested that the coexistence of MDS and plasma cell neoplasms could be explained by several mechanisms including, disruption of the bone marrow microenvironment or genetic abnormalities in hematopoietic stem cells. Klimkowska et al.,^12^ studied the genetic profiles of isolated myeloid and plasma cell compartments in 27 patients with co-existing non-chemotherapy related MDS and plasma cell neoplasm (20 patients with MGUS, 6 patients with multiple myeloma, and 1 patient with plasmacytoma). The authors did not find any evidence of founder mutations that could explain the coexistence of MDS and plasma cell neoplasms. However, they had only studied the exomes; they couldn’t exclude the possibility that there could be common genetic abnormalities outside the exomes, which could be further investigated by whole genome sequencing.

## CONCLUSION

Further studies are needed to understand better the bone marrow microenvironment and genetic abnormalities in those patients; to identify if there is a true common pathogenic pathway between MDS and plasma cell neoplasms. In addition, if there is a pathological link between those diseases, it is essential to know which one precedes the other.
